# Comparison of Surgical Outcomes of Two New Techniques Complementing Robotic Single-Site Myomectomy: Coaxial Robotic Single-Site Myomectomy vs. Hybrid Robotic Single-Site Myomectomy

**DOI:** 10.3390/jpm14040439

**Published:** 2024-04-22

**Authors:** Nara Lee, Su-Hyeon Choi, Seyeon Won, Yong-Wook Jung, Seung-Hyun Kim, Jin-Yu Lee, Chul-Kwon Lim, Jung-Bo Yang, Joong-Gyu Ha, Seok-Ju Seong

**Affiliations:** 1 Department of Obstetrics and Gynecology, CHA Gangnam Medical Center, CHA University, Seoul 06135, Republic of Korea; naradd@chamc.co.kr (N.L.); k345@chamc.co.kr (S.-H.C.); drtong85@chamc.co.kr (S.W.); dumbung@chamc.co.kr (Y.-W.J.); 2 Department of Obstetrics and Gynecology, Eulji University Hospital, Eulji University School of Medicine, 95 Dunsanseo-ro, Seo-gu, Daejeon 35233, Republic of Korea; mdearnest@eulji.ac.kr (S.-H.K.); 20220524@eulji.ac.kr (J.-Y.L.); ck7095@eulji.ac.kr (C.-K.L.); yangjb@eulji.ac.kr (J.-B.Y.)

**Keywords:** robotic surgical procedures, robotic myomectomy, robotic single-site myomectomy, uterine myomectomy, uterine fibroids, propensity score matching

## Abstract

Background: This study aimed to compare surgical outcomes between two new robotic single-site myomectomy (RSSM)-complementary techniques: coaxial robotic single-site myomectomy (Coaxial-RSSM) and hybrid robotic single-site myomectomy (Hybrid-RSSM). Methods: Medical records for 132 women undergoing Coaxial-RSSM and 150 undergoing Hybrid-RSSM, consecutively, were retrospectively reviewed. Patient characteristics and surgical outcomes were assessed and compared after propensity score matching (PSM). Results: In the outcomes of PSM, the Coaxial-RSSM group showed significantly reduced blood loss (79.71 vs. 163.75 mL, *p* < 0.001) and reduced hospital duration (4.18 ± 0.62 vs. 4.63 ± 0.90) relative to the Hybrid-RSSM group. Conversely, Hybrid-RSSM allowed for a shorter operative time compared with Coaxial-RSSM (119.19 vs. 156.01 min, *p* = 0.007). No conversions to conventional laparoscopy or laparotomy or any need for the multi-site robotic approach occurred in either group. Postoperative complications, including ileus, fever, and wound dehiscence, showed no statistically significant differences between the two groups. Conclusions: Blood loss was lower with Coaxial-RSSM, and operative time was shorter for Hybrid-RSSM. A follow-up prospective study is warranted for more comprehensive comparison of surgical outcomes between the two techniques.

## 1. Introduction

Uterine myomas, commonly known as fibroids, stand as the predominant benign neoplasm found in the female reproductive tract [1]. These growths manifest in a significant proportion of women during their fertile years, with statistics showing that fully 20–40% of reproductive-aged women are affected. The prevalence of these fibroids escalates with age, culminating in a staggering 70% incidence rate by the time women reach menopause [2,3,4,5]. Fibroids can lead to a variety of symptomatic manifestations, including heavy menstrual bleeding (menorrhagia), dysmenorrhea, anemia, pressure symptoms due to their mass effect, and potential complications that may impact pregnancy [4]. Given these potential complications, management of symptomatic fibroids often necessitates a multifaceted approach encompassing conservative management strategies, targeted symptomatic treatments, and/or surgical intervention [4,5].

Historically, hysterectomy has been the predominant treatment option for symptomatic fibroid cases [6,7]. However, recent statistical data indicate a different trend. As reported by the Korean National Health Insurance Service (NHIS), the number of individuals diagnosed with myomas has increased significantly over the years, rising from 154,080 in 2002 to 557,541 in 2016. Note that this represents a more-than-threefold increase in just fourteen years. Over a similar timespan, 2002 through 2013, the percentage of myoma patients choosing myomectomy, a fertility-preserving alternative to hysterectomy, increased from 22% to 49%. The preference for hysterectomy during the same period, conversely, saw a decline from 78 to 45%. These data not only underscore the growing awareness and diagnosis of fibroids but also highlight a shift in treatment preferences among those affected [8]. Indeed, in Korea, as the trend toward delaying childbearing in favor of careers or travel has taken hold among increasing numbers of women, more and more of those of reproductive age are seeking uterine-sparing surgery to preserve their fertility potential [3,8].

Robotic myomectomy, a type of laparoscopic myomectomy, is a minimally invasive way for surgeons to remove uterine fibroids [9,10,11]. The use of the da Vinci robot system (Intuitive Surgical., Inc., Sunnyvale, CA, USA) has become widespread in myomectomy, particularly given its advantages such as a three-dimensional surgical view, reduced surgeon’s tremor, and improved suturing capabilities. Similarly to laparoscopic myomectomy, robot-assisted laparoscopic myomectomy presents several benefits over myomectomy performed through laparotomy, such as reduced blood loss, shorter hospital stays, and fewer surgical complications [12,13]. Single-site robotic surgery offers better cosmetic results compared with multi-port robotic surgery, as it necessitates only one intra-umbilical skin incision [11,14]. Robotic single-site myomectomy (RSSM) has emerged as a promising approach, with studies highlighting its feasibility and safety relative to single-site laparoscopic myomectomy [15,16,17,18,19]. Similarly, in a comparative study of robotic single-site versus multi-port myomectomy, it was reported that da Vinci robotic single-site myomectomy is a viable option in terms of reproducibility and safety [20]. For patients requiring safe and cosmetically appealing myomectomy, RSSM may be the most suitable option. However, RSSM still has a weakness in the crucial aspect of traction power for myoma removal [21,22]. Utilization of semi-rigid instruments and the absence of traction devices are challenging aspects of myomectomy procedures using robotic single-port systems. Inserting a 5 mm tenaculum instrument through an assistant trocar can provide help with counter-traction during myoma removal, but due to the limitations of the single-site opening, there is restricted movement and increased instrument collision, which hinders proper manipulation.

To overcome this limitation, our institution has introduced two innovative techniques: Hybrid-RSSM and Coaxial-RSSM. Hybrid-RSSM is a method that combines the benefits of single-site laparoscopy and single-site robotic surgery [23]. After removing fibroids through single-site laparoscopy, the single-site robot is docked, and suturing is performed using a wristed needle holder. When employing a 5 mm tenaculum instrument with assistance from an assistant port in RSSM, a total of four instruments, including the camera, are used through a single site. However, in the conventional single-site laparoscopy system, the use of a 5 mm tenaculum instrument typically involves only three instruments, including the camera. This setup provides relatively more space and maneuverability and allows for greater freedom in adjusting the angle of counter-traction, thereby increasing traction power. In this context, Coaxial-RSSM emerged, incorporating conventional robot cannulas and instruments to a single site through various manipulations. Significantly, this enables utilization of strong, 8 mm instrument for myoma removal and suturing [22]. In previous studies, we compared both Hybrid-RSSM and Coaxial-RSSM with RSSM, respectively, and reported on the surgical outcomes [22,23]. Hybrid-RSSM reduced operative time (98.7 ± 31.7 vs. 141.4 ± 54.4 min, *p* < 0.001) and afforded a lower estimated blood loss (EBL; 131.5 ± 78.1 vs. 212.3 ± 189.8 mL, *p* < 0.001) relative to RSSM. Similarly, Coaxial-RSSM was associated with a shorter operative time (101.0 vs. 146.1 min, *p* = 0.008) and lower EBL (75.0 vs. 210.5 mL, *p* = 0.001) relative to RSSM. The objective of the present study was to compare the surgical outcomes of Hybrid-RSSM with those of Coaxial-RSSM, which both offer unique advantages as RSSM-complementing techniques.

## 2. Materials and Methods

### 2.1. Patient Population

This retrospective study was conducted at two institutions: CHA Gangnam Medical Center, CHA University and Daejeon Eulji Medical Center, Eulji University. It spanned from August 2016 to July 2023, having received ethical approval from the Institutional Review Board of CHA Gangnam Medical Center (2024-01-022). It included patients with one or more myomas requiring removal during the same period, who underwent either Hybrid-RSSM or Coaxial-RSSM. A total of 132 patients underwent Coaxial-RSSM, and 150 underwent Hybrid-RSSM. These women had been offered treatment options by their physicians. A total of 182 patients from Gangnam Cha Hospital and 100 from Daejeon Eulji Hospital were enrolled and analyzed in this study. After PSM, 91 patients from Gangnam Cha Hospital and 45 from Daejeon Eulji Hospital were included in the analyses.

### 2.2. Variables

The following medical, perioperative, and pathologic information was obtained from a database review; age, body mass index (BMI), parity, previous abdominal surgical history, peritoneal adhesion, concurrent surgery, myoma characteristics, operative time, preoperative hemoglobin (Hb), postoperative Hb, transfusion, hospital duration, and postoperative complications. Myoma characteristics were described in terms of the total number of removed myomas, the size of the largest myoma (cm), myoma weight (g), and the uterine location of the largest myoma classified as anterior, posterior, fundal-anterior, fundal-posterior, or fundal. Additionally, myoma types (types 0–8) were investigated based on the FIGO subclassifications; accordingly, the subgroups submucosal (types 1–2), intramural (types 3–5), and subserosal (types 6–8). Preoperative Hb levels were assessed within a month of the operation, and postoperative Hb levels were measured on the first day after the operation. The hemoglobin decrement (g/dL) was calculated as the difference between preoperative Hb and postoperative Hb. The difference was analyzed, excluding patients who received transfusions not only during surgery but also throughout the hospitalization period. Operative time was defined as the duration from the start of skin incision to completion of skin closure. Transfusions were performed at the discretion of the physician in cases where vital signs were unstable, hemoglobin levels were 7 or below, or EBL exceeded 500 mL. The complications were evaluated as ileus, fever, and wound dehiscence. Ileus was defined as failure to pass bowel gas after surgery and was confirmed by an abdominal X-ray showing ileus. Fever was defined as a temperature of 38 °C or higher persisting for more than 3 days, raising suspicion of infection. Wound dehiscence was assessed during the one-month postoperative period.

### 2.3. Surgical Procedure

All of the patients underwent general anesthesia with endotracheal intubation and were positioned in the dorsal lithotomy position. Subsequently, aseptic dressing and draping protocols were implemented. A Foley catheter was indwelled into the bladder, and a uterine RUMI manipulator was implemented to control the position of the uterus. An incision of 2.5–3.0 cm was made at the umbilicus for insertion of a glove port (Nelis, Seoul, Republic of Korea). Then, after the patients were placed in the Trendelenburg position, the pelvic cavities were explored.

#### 2.3.1. Hybrid-RSSM

Hybrid-RSSM entails performing myoma removal by laparoscopy followed by utilization of the robotic single-site system for suturing. After the abdomen was insufflated with CO_2_ gas, a 5 mm 30 degree rigid laparoscope was inserted and employed for exploration. A diluted solution of vasopressin (25 units of vasopressin in 100 mL of saline) was injected into the myometrium for hemostatic control. Following this, a transverse or longitudinal incision was made above the myoma. The myoma was enucleated using a harmonic scalpel (Ethicon, Somerville, NJ, USA) and grasped with a tenaculum grasper, myoma screw, or craw forceps. After enucleation, the da Vinci^®^ Technology xi or x robotic system (Intuitive Surgical, Inc.) was docked vertically, and an 8.5 mm 30 degree da Vinci stereo laparoscope was introduced. Following docking, a wristed needle holder and bipolar forceps were placed in the robotic arms. The uterine wall was sutured layer by layer with continuous suturing using a 2-0 V-Loc (Covidien, Dublin, Ireland) or 2-0 monofix (Samyang, Seoul, Republic of Korea). The peritoneum, fascia, and subcutaneous layers were closed using 1-0 vicryl (Ethicon), and the skin was closed using 3-0 vicryl sutures

#### 2.3.2. Coaxial-RSSM

The first incision of 2.0–2.5 cm was made at the umbilicus, and then a 4-channel glove port (Nelis, Seoul, Republic of Korea) was inserted into the incision. The three 8 mm robotic cannulas, including a regular-length 10 cm cannula for the camera port, a 10 cm cannula for one instrument, and a long 15 cm cannula for the other instrument, were inserted in each channel of the glove port. The patient-side cart was docked onto the three cannulas, arm 1 connected to the 10 cm cannula, arm 2 to the camera-port cannula, and arm 3 to the long cannula. In single-port robotic surgery, where all instruments need to fit through a single small hole, each cannula typically forms a triangular configuration. However, in Coaxial-RSSM, the two cannulas and the camera cannula are arranged to form a reversed triangle. Additionally, to minimize collisions between instruments, a camera scope for a 30 degree upward view was used. One of the arms was equipped with a long cannula, 15 cm in length, rather than the standard 10 cm. This positioning places it higher above the abdominal wall relative to the other arm and the camera arm. This setup ensures a sufficient distance between the two arms. Arm 1 and Arm 3 can be equipped with conventional 8 mm instruments, such as a robotic tenaculum or fenestrated bipolar forceps, and wristed monopolar, curved scissors, or the mega-needle driver, as desired. Coaxial-RSSM utilizes the conventional multi-port robotic instrument, the tenaculum, to enhance the strength of traction during myoma removal. Additionally, a mega-needle driver is employed to enhance suturing capabilities. The uterine wall was continuously sutured layer by layer with suturing using 2-0 V-Loc (Covidien, Dublin, Ireland) and 2-0 monofix (Samyang, Seoul, Republic of Korea). The peritoneum, fascia, and subcutaneous layers were closed using 1-0 vicryl (Ethicon), and the skin was closed using 3-0 vicryl sutures.

### 2.4. Statistical Analysis

Continuous variables were recorded as mean ± standard deviations. The statistical analysis entailed the Mann–Whitney U test for continuous variables and the χ^2^ test for categorical variables, with Fisher’s exact test for non-parametric statistics. To assess surgical outcomes with minimized selection bias, we conducted 1:1 propensity score matching (PSM) for age, BMI, parity, prior abdominal surgery, concurrent surgery, largest myoma size, and tumor weight between the Hybrid-RSSM and Coaxial-RSSM groups. SPSS version 24.0 (IBM Inc., Armonk, NY, USA) was used for the analyses, and statistical significance was defined as a *p* value < 0.05.

## 3. Results

### 3.1. Baseline Characteristics of Myomectomy Patients

A total of 150 women underwent Hybrid-RSSM, while 132 underwent Coaxial-RSSM. Table 1 presents the baseline clinical and surgical characteristics of all of the study patients. In the Coaxial-RSSM group, relative to Hybrid-RSSM, there were larger largest myoma size (8.14 ± 1.85 vs. 6.31 ± 1.86 cm, *p* < 0.001) and heavier tumor weight (183.85 ± 139.70 vs. 118.13 ± 95.24 cm, *p* < 0.001). Additionally, the Coaxial-RSSM group exhibited more instances of previous abdominal surgery (54 vs. 20, *p* < 0.001), higher BMI (23.06 ± 3.80 vs. 21.58 ± 2.46, *p* = 0.001), and more frequent concurrent surgery (29 vs. 21 cases, *p* < 0.001) compared with Hybrid-RSSM. Peritoneal adhesion (28, 18.7% vs. 26, 19.7%, *p* = 0.88), total myoma count (2.23 ± 1.63 vs. 3.20 ± 3.93, *p* = 0.381), myoma location, and myoma type according to the FIGO classification showed no significant differences between Hybrid-RSSM and Coaxial-RSSM.

### 3.2. Surgical Outcomes

As seen in Table 2, in the total data, Hybrid-RSSM showed shorter operative time (113.23 ± 32.85 vs. 181.09 ± 81.72 min, *p* < 0.001) relative to Coaxial-RSSM. However, Coaxial-RSSM demonstrated lower blood loss (92.42 ± 102.09 vs. 143.0 ± 102.71 mL, *p* < 0.001), reduced Hb decrement (1.11 ± 1.28 vs. 1.59 ± 0.91), and a shorter hospital stay (4.31 ± 0.87 vs. 4.59 ± 0.84, *p* = 0.001). There were no significant differences in the rates of conversion to laparotomy, or conventional laparoscopy, or in the need for an additional port, between the two groups (*p* = 0.468). In the Hybrid-RSSM group, there were two patients (1.3%) who received conservative treatment for postoperative ileus, while in the Coaxial-RSSM group, there were five such patients (3.8%). Only one patient in the Hybrid-RSSM group experienced fever lasting more than 3 days, whereas there were no such cases in the Coaxial-RSSM group. There were no cases of wound dehiscence reported for the Hybrid-RSSM group, while one patient in the Coaxial-RSSM group experienced wound dehiscence. However, postoperative complications including ileus, fever > 3 days, and wound dehiscence showed no significant differences between the two groups (*p* = 0.173).

### 3.3. Propensity Score Matching (PSM) Analysis

Table 3 displays the patients’ baseline characteristics after propensity score matching (PSM). There were no intergroup differences in terms of mean age (*p* = 0.138) or BMI (*p* = 0.739). However, the mean parity (0.37 ± 0.64 vs. 0.71 ± 0.93, *p* = 0.046) was significantly different. In both groups, intramural (types 3–5) was the most common type, with 54 cases in the Hybrid-RSSM group accounting for 79.4% and 60 cases in Coaxial-RSSM for 88.2% (*p* = 0.177). Myoma type, myoma number (2.04 ± 1.30 vs. 3.32 ± 4.15, *p* = 0.937), largest size of myoma (7.40 ± 1.64 vs. 7.60 ± 1.34, *p* = 0.447), and myoma weight (163.26 ± 105.81 vs. 180.08 ± 135.68, *p* = 0.829) showed no significant intergroup differences.

As indicated in Table 4, Hybrid-RSSM still showed a shorter operative time (119.19 ± 30.54 vs. 156.01 ± 72.49 min, *p* = 0.007) compared with Coaxial-RSSM. However, Coaxial-RSSM maintained lower blood loss (79.71 ± 88.49 vs. 163.75 ± 127.67 mL, *p* < 0.001) and a shorter hospital stay (4.18 ± 0.62 vs. 4.63 ± 0.90, *p* = 0.001) relative to Hybrid-RSSM. Regarding Hb decrement, no significant intergroup differences were observed (1.53 ± 0.92 vs. 1.33 ± 0.09, *p* = 0.229). After PSM, there were no cases of conversion to laparotomy, or conventional laparoscopy, or any need for an additional port in either group. In the Hybrid-RSSM group, one patient (1.5%) received conservative treatment for postoperative ileus, while two in the Coaxial-RSSM group did so (2.9%). Among the patients, one in the Hybrid-RSSM group experienced fever lasting more than 3 days, whereas there were no such cases in the Coaxial-RSSM group. Neither group reported any instances of wound dehiscence. Postoperative complications including ileus, fever >3 days, and wound dehiscence showed no significant intergroup differences either before or after PSM.

## 4. Discussion

Myomectomy usually requires multiple intra-corporeal sutures, and accessing uterine myomas laparoscopically can be challenging due to their varied sizes and types, even with the assistance of robotic surgical systems. RSSM has the limitation of relatively weak counter-traction power during myoma removal. To overcome this, two techniques, Hybrid-RSSM and Coaxial-RSSM, have been devised, each with its advantages. Hybrid-RSSM excels in providing sufficient traction for the removal of deep myomas using a 5 mm myoma screw and tenaculum in the laparoscopy. Moreover, the use of advanced bipolar devices or ultrasonic scalpels contributes to reduction in both total operative time and blood loss. On the other hand, Coaxial-RSSM capitalizes on a conventional rigid robot system, utilizing an 8 mm tenaculum to enhance traction strength. Unlike RSSM and Hybrid-RSSM, which use a 5 mm semi-rigid instrument, Coaxial-RSSM employs an 8 mm mega-needle driver, thus affording stronger suturing strength.

The main findings of this study were that the operative time was shorter for Hybrid-RSSM while the EBL was lower for Coaxial-RSSM. In PSM, Hybrid-RSSM showed a shorter operative time than did Coaxial-RSSM (119.19 ± 30.54 vs. 156.01 ± 72.49 min, *p* = 0.007). Coaxial-RSSM continued to demonstrate a lower EBL (79.71 ± 88.49 vs. 163.75 ± 127.67 mL, *p* < 0.001) and a shorter hospital stay (4.18 ± 0.62 vs. 4.63 ± 0.90, *p* = 0.001).

After PSM, there were significant differences between the two groups in terms of parity in the baseline characteristics. Movilla et al. published research findings on the variables that prolong total operative time for robot-assisted laparoscopic myomectomy. According to that study, patient age, underlying diabetes mellitus, uterine volume, number of myomas, number of myomas larger than 3 cm, and size of largest myoma are preoperative factors significantly associated with surgical time. However, parity was reported to have no association with total operative time [24].

The difference in operative time between the two groups may be attributed to the following factors. Initially, the use of advanced bipolar or harmonic devices in the Hybrid-RSSM approach is likely to reduce the time required for myoma removal relative to Coaxial-RSSM, which uses mono-polar curved scissors. According to an RCT study comparing a group using a harmonic device with a group using epinephrine and electro-surgery devices for laparoscopic myomectomy, there was a significant difference in operative time (71.8 ± 26.7 vs. 88.8 ± 35.5 min, *p* < 0.001) [25]. However, it is important to note that that study compared conventional laparoscopic bipolar and harmonic devices, and there may be differences in the functionality between conventional laparoscopic bipolar and robotic bipolar instruments. Secondly, after PSM, there were no significant differences in the total number of myomas, largest myoma size, or tumor weight between the two groups. However, in the Coaxial-RSSM group, there were relatively more myomas, larger myoma size, and heavier myomas. Movilla et al. previously found that uterine volume, number of myomas, number of myomas > 3 cm, and diameter of dominant myoma were all associated with increased total operative time [24]. This might be another factor influencing the difference in operative time between the two groups. Thirdly, Coaxial-RSSM involves additional maneuvers during robot docking, such as forming a reverse triangle, using a long canula, and flipping the camera direction 30 degrees upside. These actions may potentially extend the time required for the robot docking process.

The main drawback of Hybrid-RSSM is that after myoma enucleation, bleeding control is not performed, and the robotic system needs to be docked. In this system, then, continuous bleeding may occur during the transition from laparoscopy to the robot mode, and this situation could potentially be associated with higher blood loss relative to Coaxial-RSSM. In fact, among the results of this study, the lower blood loss with Coaxial-RSSM (79.71 ± 88.49 vs. 163.75 ± 127.67 mL, *p* < 0.001) suggests a significant drawback to Hybrid-RSSM.

As far as we know, this report is the first to evaluate the surgical outcomes of Hybrid-RSSM compared with those of Coaxial-RSSM. Our study’s results align with the respective advantages of the two techniques, Hybrid-RSSM and Coaxial-RSSM. However, it has two limitations. Firstly, it is primarily a retrospective study with a relatively small sample size. By performing PSM, we endeavored to minimize the inherent bias associated with a retrospective design. Secondly, no detailed analysis of operative time was performed. Measuring and comparing the times required for docking, myoma removal, and suturing would have allowed a more direct comparison of the difference in surgical time between Hybrid-RSSM and Coaxial-RSSM.

## 5. Conclusions

In conclusion, this study compared the surgical outcomes of Hybrid-RSSM with those of Coaxial-RSSM, demonstrating distinct advantages for each technique. Hybrid-RSSM showed a shorter operative time, while Coaxial-RSSM was associated with lower blood loss and shorter hospital stays. For validation of these advantages within a more comprehensive overall comparison, further prospective studies are warranted.

## Figures and Tables

**Table 1:** Baseline characteristics of myomectomy patients.

Characteristics	Hybrid-RSSM (*n* = 150)	Coaxial-RSSM (*n* = 132)	*p*
Age, years	37.21 ± 5.10	41.62 ± 6.47	<0.001
BMI, kg/m^2^	21.58 ± 2.46	23.06 ± 3.80	0.001
Parity	0.37 ± 0.69	1.14 ± 1.03	<0.001
Previous abdominal surgery			<0.001
No	130 (86.7)	78 (59.1)	
Yes	20 (13.3)	54 (40.9)	
Peritoneal adhesion			0.88
No	122 (81.3)	106 (80.3)	
Yes	28 (18.7)	26 (19.7)	
Concurrent surgery			<0.001
No	129 (86.0)	103 (78.0)	
Ovarian cystectomy	20 (13.3)	9 (6.8)	
USO	0 (0)	1 (0.8)	
Focal adenomyomectomy	1 (0.7)	19 (14.4)	
Total myoma, n	2.23 ± 1.63	3.20 ± 3.93	0.381
Largest myoma size, cm	6.31 ± 1.86	8.14 ± 1.85	<0.001
Location			0.852
Anterior	75 (50.0)	75 (56.8)	
Posterior	58 (38.7)	33 (25.0)	
Fundal	10 (6.7)	18 (13.6)	
Anterior fundal	3 (2.0)	5 (3.8)	
Posterior fundal	4 (2.7)	1 (0.8)	
Type (FIGO classification)			0.752
Submucosal (types 1–2)	11 (7.3)	1 (0.8)	
Intramural (types 3–5)	112 (74.7)	118 (89.4)	
Subserosal (types 6–8)	27 (18.0)	13 (9.8)	
Tumor weight, g	118.13 ± 95.24	183.85 ± 139.70	<0.001

Note: Values are presented as number (%), median (range), or mean ± standard deviations. Abbreviations: BMI, body mass index; USO, unilateral salpingo-oophorectomy; FIGO, International Federation of Gynecology and Obstetrics; Hybrid-RSSM, hybrid robotic single-site myomectomy; Coaxial-RSSM, coaxial robotic single-site myomectomy; RSSM, robotic single-site myomectomy.

**Table 2:** Surgical outcomes and morbidity.

	Total Data		
Characteristics	Hybrid-RSSM (*n* = 150)	Coaxial-RSSM (*n* = 132)	*p*
Operative time, mins	113.23 ± 32.85	181.09 ± 81.72	<0.001
EBL, mL	143.90 ± 102.71	92.42 ± 102.09	<0.001
Hemoglobin decrement, g/dL	(*n* = 147) 1.69 ± 0.84	(*n* = 119) 1.22 ± 1.16	0.001
	1.59 ± 0.91	1.11 ± 1.28	<0.001
Transfusion			0.008
No	147 (98.0)	119 (90.2)	
Yes	3 (2.0)	13 (9.8)	
Hospital stay, days	4.59 ± 0.84	4.31 ± 0.87	0.001
Conversion			0.468
No	150 (100)	131 (99.2)	
Laparotomy	0 (0)	0 (0)	
Laparoscopy	0 (0)	1 (0.8)	
Multi-site	0 (0)	0 (0)	
Complications			0.173
None	147 (98.0)	126 (95.5)	
Ileus	2 (1.3)	5 (3.8)	
Fever > 3 days	1 (0.7)	0 (0)	
Wound dehiscence	0 (0)	1 (0.8)	

Note: Values are presented as number (%), median (range), or mean ± standard deviations. Abbreviations: EBL, estimated blood loss; Hybrid-RSSM, hybrid robotic single-site myomectomy; Coaxial-RSSM, coaxial robotic single-site myomectomy; RSSM, robotic single-site myomectomy.

**Table 3:** Baseline characteristics of myomectomy patients after propensity score matching (PSM).

Characteristics	Hybrid-RSSM (*n* = 68)	Coaxial-RSSM (*n* = 68)	*p*
Age, years	38.03 ± 4.84	39.34 ± 6.76	0.138
BMI, kg/m^2^	21.95 ± 2.56	22.12 ± 2.71	0.739
Parity	0.37 ± 0.64	0.71 ± 0.93	0.046
Previous abdominal surgery			0.836
No	52 (76.5)	54 (79.4)	
Yes	16 (23.5)	14 (20.6)	
Peritoneal adhesion			>0.999
No	57 (83.8)	58 (85.3)	
Yes	11 (16.2)	10 (14.7)	
Concurrent surgery			>0.999
No	62 (91.2)	62 (91.2)	
Ovarian cystectomy	5 (7.4)	5 (7.4)	
USO	1 (1.5)	1 (1.5)	
Focal adenomyomectomy	0 (0)	0 (0)	
Total myoma, n	2.04 ± 1.30	3.32 ± 4.15	0.937
Largest myoma size, cm	7.40 ± 1.64	7.60 ± 1.34	0.447
Location			0.794
Anterior	30 (44.1)	36 (52.9)	
Posterior	27 (39.7)	17 (25.0)	
Fundal	6 (8.8)	10 (14.7)	
Anterior fundal	3 (4.4)	4 (5.9)	
Posterior fundal	2 (2.9)	1 (1.5)	
Type (FIGO classification)			0.177
Submucosal (types 1–2)	1 (1.5)	1 (1.5)	
Intramural (types 3–5)	54 (79.4)	60 (88.2)	
Subserosal (types 6–8)	13 (19.1)	7 (10.3)	
Tumor weight, g	163.26 ± 105.81	180.08 ± 135.68	0.829

Note: Values are presented as number (%), median (range), or mean ± standard deviations. Abbreviations: BMI, body mass index; USO, unilateral salpingo-oophorectomy; FIGO, International Federation of Gynecology and Obstetrics; Hybrid-RSSM, hybrid robotic single-site myomectomy; Coaxial-RSSM, coaxial robotic single-site myomectomy; RSSM, robotic single-site myomectomy.

**Table 4:** Surgical outcomes and morbidity after propensity score matching (PSM).

	In PSM Data		
Characteristics	Hybrid-RSSM (*n* = 68)	Coaxial-RSSM (*n* = 68)	*p*
Operative time, mins	119.19 ± 30.54	156.01 ± 72.49	0.007
EBL, mL	163.75 ± 127.67	79.71 ± 88.49	<0.001
Hemoglobin decrement, g/dL	(*n* = 65) 1.53 ± 0.92	(*n* = 66) 1.33 ± 1.09	0.229
	1.46 ± 1.05	1.27 ± 1.14	0.225
Transfusion			>0.999
No	65 (95.6)	66 (97.1)	
Yes	3 (4.4)	2 (2.9)	
Hospital stay, days	4.63 ± 0.90	4.18 ± 0.62	0.001
Conversion			NS
No	68 (100)	68 (100)	
Laparotomy	0 (0)	0 (0)	
Laparoscopy	0 (0)	0 (0)	
Multi-site	0 (0)	0 (0)	
Complications			0.634
None	66 (97.1)	66 (97.1)	
Ileus	1 (1.5)	2 (2.9)	
Fever > 3 days	1 (1.5)	0 (0)	
Wound dehiscence	0 (0)	0 (0)	

Note: Values are presented as number (%), median (range), or mean ± standard deviations. Abbreviations: EBL, estimated blood loss; Hybrid-RSSM, hybrid robotic single-site myomectomy; Coaxial-RSSM, coaxial robotic single-site myomectomy; RSSM, robotic single-site myomectomy.

## Data Availability

Data are contained within the article.
